# Health-related quality of life of mothers and developmental characteristics of very low birth weight children at 2.5 years of age: results from the Japan Environment and Children’s Study (JECS)

**DOI:** 10.1186/s12955-023-02156-4

**Published:** 2023-07-10

**Authors:** Hatoko Sasaki, Kyongsun Pak, Hidetoshi Mezawa, Kiwako Yamamoto-Hanada, Kazue Ishitsuka, Mizuho Konishi, Minaho Nishizato, Miori Sato, Mayako Saito-Abe, Limin Yang, Yukihiro Ohya, the Japan Environment, Children’s Study (JECS) Group

**Affiliations:** 1 Medical Support Center for the Japan Environment and Children’s Study, National Center for Child Health and Development, 2-10-1 Okura, Setagaya-Ku, Tokyo, 157-8535 Japan; 2 Shizuoka Graduate University of Public Health, 4-27-2 Kita-Ando, Aoi-Ku, Shizuoka-Shi, 420-0881 Japan; 3 Division of Biostatistics, Clinical Research Center, National Center for Child Health and Development, 2-10-1 Okura, Setagaya-Ku, Tokyo, 157-8535 Japan; 4 Department of Social Medicine, National Center for Child Health and Development, 2-10-1 Okura, Setagaya-Ku, Tokyo, 157-8535 Japan; 5 Department of Clinical Psychology, Tokyo Seitoku University, 1-7-13 Jujodai, Kita-Ku, Tokyo, 114-0033 Japan

**Keywords:** Health-related quality of life, Child development, Very low birth weight, Japan Environment and Children’s Study (JECS)

## Abstract

**Background:**

The level of child development may be associated with the risk of poor maternal health-related quality of life (HRQoL). The objective of this study was to describe the developmental characteristics of very low birth weight (VLBW) children at 2.5 years of age and to examine associations between maternal HRQoL and the degree of child development based on the Japanese version of Ages and Stages Questionnaire (J-ASQ-3).

**Methods:**

A cross-sectional study was performed using the data from a nationwide prospective birth cohort study in Japan. Among a total of 104,062 fetal records, the VLBW infants (birth weight ≤ 1500 g) were analyzed using linear regression models, adjusted for potential covariates. Subgroup analysis was also conducted to assess the association between social connection or cooperation of the partner and maternal HRQoL by the level of child development.

**Results:**

The final study subjects included 357 VLBW children and mothers. The suspected developmental delays (SDDs) in at least two domains was significantly associated with lower maternal mental HRQoL regression coefficient –2.314 (95%CI: –4.065 to –0.564). There was no association between the status of child development and maternal physical HRQoL. After adjusting for child and maternal covariates, the maternal HRQoL was not significantly associated with child development. Amongst women who indicated having some social support, having a child with a SDD in two or more domains was negatively associated with mental HRQoL compared with women whose child was less developmental delay, regression coefficient –2.337 (95%CI: –3.961 to –0.714). Amongst women who indicated having partner’s cooperation to child-rearing, having a child with a SDD in two or more domains was negatively associated with mental HRQoL compared with women whose child was less developmental delay, regression coefficient –3.785 (95%CI: –6.647 to –0.924).

**Conclusions:**

Our findings indicate that the lower maternal mental HRQoL was independently associated with the SDDs evaluated by the J-ASQ-3, whereas there was no association after adjusting for covariates. Further research is warranted to elucidate the impact of social connection and partner’s cooperation on maternal HRQoL and child development. This study urges that particular attention should be paid to mothers of VLBW children with SDDs and also to provide early intervention and continued support.

**Supplementary Information:**

The online version contains supplementary material available at 10.1186/s12955-023-02156-4.

## Background

The birth rates of very low birth weight (VLBW) infants in Japan have remained at 0.7% to 0.8% of newborn babies over the past decade [1]. The Neonatal Intensive Care Unit (NICU) mortality rate, excluding post-discharge mortality, declined over time from 10.7% in 2003 to 5.2% in 2015, and the mortality rate by the age of 3 years decreased from 11.9% in 2003 to 5.6% in 2015 [2]. Although more VLBW infants are surviving, they have potential risks of morbidity [3], neurodevelopmental outcomes [4], and continuing healthcare.

A recent meta-analysis of 30 studies from various countries [4] reported that the prevalence of cognitive and motor delays in VLBW infants from 18 months to 3 years, evaluated with the Bayley scale (Bayley Scale of Infant Development), was estimated at 16.9% (95% confidence interval [CI]: 10.4% to 26.3%) and 20.6% (95%CI: 13.9% to 29.4%), respectively. In Japan, 16.3% of VLBW infants born between 2003 and 2015 had developmental delay at 3 years of age evaluated with the Kyoto Scale of Psychological Development (KSPD) test, which is a standardized and validated development assessment for Japanese children [2]. Both Bayley and KSPD are completed by either physicians or trained psychologists. Although the results of the two tests are not directly comparable, the development characteristics of the motor, cognitive, and language scores on the latest version of KSPD were well correlated with those on Bayley III [5]. Although the percentage of developmental delay in Japanese VLBW children at 3 years of age seems a little less than the synthesized prevalence of meta-analysis as indicated above, it is not a small number that 16.3% of Japanese VLBW children have developmental delays at 3 years of age.

The Ages and Stages Questionnaire (ASQ-3) is a valid developmental screening measure completed by parents or caregivers and was originally developed to monitor premature infants discharged from the NICU [6]. The ASQ-3 has been shown to be feasible with a high response rate [7] and be cost-effective [8] for screening children who may require further developmental assessment in a clinical setting. The ASQ-3 seems to be suitable for monitoring child development of VLBW children in a large population as compared with other measures requiring trained personnel. The benefit of assessment tools such as ASQ-3 is that it is useful for parents and caregivers to have active engagement with their child’s development.

An unbalanced development between the areas of cognition, language, and movement is common in preterm and VLBW infants [9]. Mansson et al. [10] reported that extremely preterm children at 2.5 years show significant lower cognitive, communicative and motor function levels compared with children born at term. Parenting VLBW children with such developmental features, particularly for mothers who are the primary caregivers, can increase difficulty and anxiety, and may therefore impact their health-related quality of life (HRQoL). Mothers of VLBW children in the USA have reported lower scores of their HRQoL compared with mothers of term children during the postpartum period [11], during the NICU hospitalization [12], and at 5 years of age [13], although one study found that HRQoL of primary caregivers of VLBW infants at 12–19 months did not differ between the full-term birth and pre-term birth groups [14]. However, to the best of the authors’ knowledge, no study has evaluated whether HRQoL among VLBW mothers is associated with child development and differs depending on the level of the child’s development.

In Japan, parents raising children, especially in urban areas, are often in nuclear families with both parents working. If there are no grandparents or other relatives in the neighborhood, they are more likely to feel burdened and isolated in raising their children. Social connection is recognized as one of the important factors for parental psychological wellbeing of child rearing. Women with low social connection are more likely to suffer from postpartum depression [15] and to report lower HRQoL [16] than well-supported women. Lower levels of social connection have also been shown to be negatively associated with higher levels of parenting stress in mothers with preterm or VLBW infants [17–20]. The perceived social connection may independently impact HRQoL among mothers with VLBW children.

The aim of this study was to describe the developmental characteristics of VLBW children at 2.5 years of age and to examine associations between maternal HRQoL and the level of child development based on the J-ASQ-3. We hypothesized that SDDs in VLBW children are negatively associated with maternal HRQoL. We also hypothesized that feeling lack of social connection and/or feeling lack of support from partners are associated with the lower maternal HRQoL, regardless of the level of child development.

## Methods

### Participants

The Japan Environment and Children’s Study (JECS) is a prospective nationwide birth cohort study that was founded by the Japanese Ministry of the Environment. The JECS covers a wide geographical area of Japan and comprises fifteen Regional Centers (Hokkaido, Miyagi, Fukushima, Chiba, Kanagawa, Koshin, Toyama, Aichi, Kyoto, Osaka, Hyogo, Tottori, Kochi, Fukuoka, and South Kyushu/Okinawa). Participants are pregnant women and their partners who were recruited during their early pregnancy from co-operating health care providers or local government offices where the Maternal and Child Health Handbook was provided. The main aim of the JECS is to evaluate whether environmental factors, such as chemicals, physical activity, and lifestyle affect childhood health. The health of mothers reflects genetic factors and lifestyle, and one of the important themes of the study is to establish how the health of mothers during pregnancy affects the subsequent health of their children. Recruitment began in January 2011, and the number of pregnant women enrolled reached 100,000 in March 2014 [21, 22]. Participating children are expected to remain in the study until they reach 13 years of age. This study was registered in the UMIN Clinical Trials Registry (number: UMIN000030786) on 12/01/2018. The present study used the dataset jecs-ta-201901930-qsn, which was released in October 2019 and revised in February 2020.

The eligibility criteria for participants in the JECS were as follows: 1) the participant should reside in the study area at the time of recruitment and expect to continue to reside in Japan for the foreseeable future; 2) their expected delivery date should be between 1 August 2011 and mid-2014; and 3) the participant should be capable of participating in the study without difficulty, i.e., they must be able to understand the Japanese language and complete the self-administered questionnaire. The study population contained 104,062 fetal records. Among them, the VLBW infants (birth weight ≤ 1500 g) regardless of gestational age are study subjects in the present study. This study simply focused on birth weight and the difficulty in raising children born small.

### Assessment of maternal HRQoL

The maternal HRQoL (health-related quality of life) was assessed by the Japanese version of the SF-8 [23] when VLBW children were at 2.5 years old. The SF-8 is a self-completed questionnaire with eight subscales, which are condensed into two summary scores, namely, physical and mental HRQoL. Scores range from 0 to 100, with higher scores indicating better HRQoL. The standardised values of scores based on national norms (50) are calculated for physical and mental HRQoL, with scores lower than 50 indicating lower HRQoL than the average Japanese. By gender, the standard values for Japanese women are slightly lower than for Japanese men [23].

### Assessment of child development

The developmental characteristics of VLBW children were assessed by the Japanese version of the Ages and Stages Questionnaire, 3^rd^ edition (J-ASQ-3) [24] at 2.5 years old in the JECS. The J-ASQ-3 is parent completed and widely used to measure developmental milestones. The questionnaire includes five developmental domains to assess: 1) Communication, 2) Gross motor skills, 3) Fine motor skills, 4) Problem-solving ability, and 5) Personal/social skills. The total score ranges from 0 to 60 by domain. We used the J-ASQ-3 cut-off points for 2.5 years old calculated by using the entire database of the JECS population to categorize the following three groups of child development: Normal, Monitoring, and Needs assessment. We used J-ASQ-3 scores corresponding to the applicable children’s ages at the completion of the questionnaire for 2.5 years, 28 months 16 days to 31 months 15 days. In the case of preterm infants, the modified age in months was calculated from the expected date of delivery and whether the modified age in months was included in the indicated age in months. When we examined the normality of the scores in our population, we found non-normality. Therefore, we reported a percentile cutoff, which is appropriate for dealing with non-normality data in addition to the SD cutoff used in many studies. In this study, we used the percentile cut-off points (above 16.0 percentile, 2.5–16.0 percentile, below 2.5 percentile) for primary analysis and the mean SD cut-off points (above 1 SD below the mean, between 1–2 SD below the mean, below 2 SD below the mean) for secondary analysis. Summaries of categorical J-ASQ-3 using the percentile cut-off points and the mean SD cut-off points are shown in Supplementary Table 1 and Supplementary Table 2, respectively.

**Table 1:** Characteristics of mothers and children (*N* = 357)

Characteristic	Category	Summary Statistics	Missing, No. (%)
**Mothers**			
Age, mean (SD)		35.2 (5.3)	0 (0.0%)
Highest educational level, No. (%)	< 9y	10 (2.8%)	37 (10.4%)
	9-12y	105 (29.4%)	
	13-14y	133 (37.3%)	
	> = 15y	72 (20.2%)	
Gestational age, mean (SD)		29.2 (3.2)	0 (0.0%)
Marital status, No. (%)	Married	338 (94.7%)	10 (2.8%)
	Divorced	5 (1.4%)	
	Bereaved	0 (0.0%)	
	Other	4 (1.1%)	
Psychological stress, No. (%)	No (K6 < 5)	278 (77.9%)	9 (2.5%)
	Yes (K6 > = 5)	70 (19.6%)	
Suspicion of mental illness, No. (%)	No (K6 < 13)	339 (95.0%)	9 (2.5%)
	Yes (K6 > = 13)	9 (2.5%)	
Cooperation of partner, No. (%)	Never	9 (2.5%)	11 (3.1%)
	Seldom	13 (3.6%)	
	Sometimes	92 (25.8%)	
	Always	232 (65.0%)	
Someone to talk to about parenting, No. (%)	No	14 (3.9%)	10 (2.8%)
	Yes	333 (93.3%)	
Stress event, No. (%)	No	223 (62.5%)	22 (6.2%)
	Yes	112 (31.4%)	
Social Connection 1, No. (%)	None	15 (4.2%)	0 (0.0%)
	Sometimes	38 (10.6%)	
	Some extent	102 (28.6%)	
	Most times	36 (10.1%)	
	Always	166 (46.5%)	
Social Connection 2, No. (%)	None	10 (2.8%)	2 (0.6%)
	Sometimes	44 (12.3%)	
	Some extent	78 (21.8%)	
	Most times	42 (11.8%)	
	Always	181 (50.7%)	
Social Connection 3, No. (%)	None	11 (3.1%)	0 (0.0%)
	Rarely	61 (17.1%)	
	Some extent	127 (35.6%)	
	Most times	56 (15.7%)	
	Always	102 (28.6%)	
Social Connection 4, No. (%)	None	8 (2.2%)	0 (0.0%)
	1 ~ 3	193 (54.1%)	
	> = 4	156 (43.7%)	
※ Sum of the Score of Social Connection 1–4, No. (%)	0	7 (2.0%)	2 (0.6%)
	1	24 (6.7%)	
	2	16 (4.5%)	
	3	19 (5.3%)	
	4	46 (12.9%)	
	5	29 (8.1%)	
	6	18 (5.0%)	
	7	16 (4.5%)	
	8	33 (9.2%)	
	9	34 (9.5%)	
	10	55 (15.4%)	
	11	58 (16.2%)	
Mother’s Physical QOL at 2.5 y.o., mean (SD)		48.601 (7.552)	0 (0.0%)
Mother’s Mental QOL at 2.5 y.o., mean (SD)		48.593 (6.224)	0 (0.0%)
**Children**			
Gender of the child, No. (%)	Male	175 (49.0%)	0 (0.0%)
	Female	182 (51.0%)	
Medical problems of the child, No. (%)	No	220 (61.6%)	23 (6.4%)
	Yes	114 (31.9%)	
Duration of hospitalization of child, Median days (IQR)		74.0 (53.0–99.0)	86 (24.1%)

**Table 2:** Univariable regression using percentile cut-off points for association between maternal HRQoL and development of VLBW children at 2.5 years old

Variable	Analysis Dataset No	Group	No. (%)	Physical QOL Coefficient (95% CI)	Mental QOL Coefficient (95% CI)
Communication	356	Above 16.0 percentile [Reference]	197 (55.3%)	-	-
		Between 2.5–16.0 percentile	117 (32.9%)	-0.981 (-2.710—0.749)	-0.394 (-1.818—1.030)
		Below 2.5 percentile	42 (11.8%)	-1.245 (-3.764—1.273)	-1.672 (-3.745—0.401)
Gross motor	356	Above 16.0 percentile [Reference]	183 (51.4%)	-	-
		Between 2.5–16.0 percentile	116 (32.6%)	-0.941 (-2.702—0.819)	-0.717 (-2.154—0.720)
		Below 2.5 percentile	57 (16.0%)	-0.278 (-2.528—1.972)	-2.683 (-4.520—-0.847)
Fine motor	353	Above 16.0 percentile [Reference]	201 (56.9%)	-	-
		Between 2.5–16.0 percentile	117 (33.1%)	-0.625 (-2.355—1.105)	-0.568 (-1.976—0.840)
		Below 2.5 percentile	35 (9.9%)	-0.201 (-2.925—2.524)	-3.667 (-5.884—-1.449)
Problem- solving	356	Above 16.0 percentile [Reference]	205 (57.6%)	-	-
		Between 2.5–16.0 percentile	93 (26.1%)	-0.670 (-2.526—1.186)	-1.129 (-2.638—0.380)
		Below 2.5 percentile	58 (16.3%)	-0.612 (-2.820—1.596)	-2.803 (-4.598—-1.008)
Personal- social	357	Above 16.0 percentile [Reference]	216 (60.5%)	-	-
		Between 2.5–16.0 percentile	89 (24.9%)	0.242 (-1.624—2.107)	-1.148 (-2.683—0.386)
		Below 2.5 percentile	52 (14.6%)	-1.304 (-3.592—0.983)	-1.118 (-3.000—0.764)
Overall	352	No domain below 2.5 percentile [Reference]	253 (71.9%)	-	-
		Any domain below 2.5 percentile	99 (28.1%)	-0.217 (-1.980—1.546)	-1.592 (-3.039—-0.145)
Overall (2 domains)	350	One or no domain below 2.5 percentile [Reference]	292 (83.4%)	-	-
		At least 2 domains below 2.5 percentile	58 (16.6%)	-1.104 (-3.243—1.035)	-2.314 (-4.065—-0.564)

### Covariates

The mother’s social connection was collected using a self-administered questionnaire at 2.5 years old. There were four items assessing the mother’s recognition of having social connection, as follows: 1) Do you have a person who is contactable and shows you love and concern? (None; Sometimes; Some extent; Most times; Always); 2) Do you have a person who supports you mentally and can help you with any issues or make difficult decisions? (None; Sometimes; Some extent; Most times; Always); 3) Are you in touch with trusted people you feel close to when you want them? (None; Rarely; Some extent; Most times; Always); 4) How many relatives and friends do you feel free to consult with? (None; 1–3; ≥ 4).

The following mother’s information was also collected using a self-administered questionnaire: highest educational level (< 9y; 9-12y; 13-14y; ≥ 15y) during pregnancy, marital status (Married; Divorced; Bereaved; Other) at 6 months, cooperation of partner (Never; Seldom; Sometimes; Always) and someone to talk to about parenting (No; Yes) at 2 years old, maternal age and stress event (No; Yes) at 2.5 years old. The maternal mental health at 2 years old was assessed by the Japanese version of Kessler Psychological Distress Scale (K6) [25]. The K6 is intended to be used as a quick tool to assess risk for serious mental illness in the general population. The K6 is a 6-item self-report measure of psychological distress in the past month, with each question answered on a five-point scale, from 0 (low stress) to a maximum of 24 (high stress) [26]. The cut-offs of psychological stress (K6 < 5; K6 ≥ 5) [27] and suspicion of mental illness (K6 < 13; K6 ≥ 13) [28] were used. The information on gestational age, gender of the child, and duration of hospitalization of child (days) were obtained using medical transcripts at birth. The information on congenital anomalies of the child (No; Yes) was transcribed from medical records at the first month check-up.

### Analytic approach

Mean and standard deviation (SD) (for normally distributed data) or median and range (for skewed data) for the maternal HRQoL, child development, and other background factors were calculated. Univariable and multivariable linear regression analyses were conducted to evaluate the association between child development and maternal physical or mental HRQoL, controlling for covariates. Missing responses of covariates were handled with multivariate imputation to compare the results of complete cases of the analyses. Subgroup analysis was also performed to assess the association between child development and maternal physical or mental HRQoL by the presence or absence of “social connection” or “cooperation of partner”. The total score of the four variables of “social connection” was 0 when it is 0, and ≥ 1 when it was greater than 0. The variable of “cooperation of partner” was categorized as Yes (Always) or No (Never; Seldom; Sometimes). The interaction was also examined in a multivariate model including confounding factors. All analyses were conducted using R Version 3.6.2.

## Results

The final study subjects included 357 VLBW children and mothers. The mean gestational age was 29.2 ± 3.2 weeks. A total of 31.9% of children had congenital anomalies at the first month of life. The mean maternal age was 35.2 ± 5.3. A total of 19.6% of mothers reported psychological stress (K6 ≥ 5). A total of 65.0% of mothers indicated that they always received their partner’s cooperation. Asked about their social connections, between 2.2 and 4.2% of mothers replied ‘non’ to one or more of the four items. Between 10.6% to 54.1% of mothers who reported very little social connection. Mothers who answered ‘always’ on one of the four social connections items ranged from 28.6% to 50.7%. The mean mother’s physical HRQoL score and mother’s mental HRQoL score were 48.601 ± 7.552 and 48.593 ± 6.224, respectively. The descriptive characteristics of mothers and children are shown in Table 1.

### Suspected developmental delay of VLBW children

**Fig. 1:**
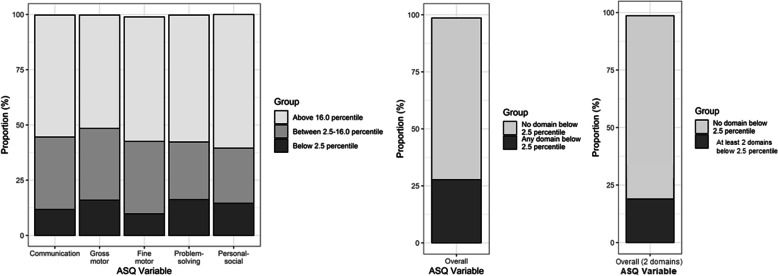
Percentages of suspected developmental delay in J-ASQ-3 domains

Individual domain-specific prevalence of delay (below 2.5 percentile) was observed as scores < 15 (11.8%) in Communication, scores < 30 (16.0%) in Gross motor, scores < 15 (9.8%) in Fine motor, scores < 20 (16.2%) in Problem solving, and scores < 25 (14.6%) in Personal social domain. A total of 27.7% of children had a delay in at least one of five domains and 16.2% of children showed a delay in at least two out of five domains (Fig. 1). Individual domain specific prevalence of delay (below 2 SD below the mean) was observed as scores < 29.43 (24.1%) in Communication, scores < 37.57 (29.7%) in Gross motor, scores < 21.71 (24.4%) in Fine motor, scores < 26.54 (27.5%) in Problem solving, and scores < 29.87 (22.7%) in Personal social domain. A total of 49.6% of children showed a delay in at least one out of five domains and 30.3% of children showed a delay in at least two out of five domains (see Additional file 1).

### Association between maternal HRQoL and development of VLBW children at 2.5 years old

The results of the univariable regression analysis reveal that the presence of SDDs (below 2.5 percentile) was significantly associated with lower maternal mental HRQoL in all domains except for the Communication and Personal-social domains (Table 2). A SDD in any one domain or in two or more domains was significantly associated with a lower maternal mental HRQoL (regression coefficients –1.592 (95%CI: –3.039 to –0.145) and–2.314 (95%CI: –4.065 to –0.564)), respectively. There was no association between the status of child development and maternal physical HRQoL. The results of the univariable regression analysis using mean SD cut-off points were almost the same as percentile cut-off points. SDDs (below 2 SD below the mean) in two or more domains were significantly associated with lower mental HRQoL (regression coefficient –2.260 (95%CI: –3.670 to –0.849)) but there was no such association between SDD in one domain and mental HRQoL (Table 3). After adjusting for child and maternal covariates, the maternal HRQoL was not significantly associated with child development in both percentile and SD cut-offs (Tables 4 and 5). The same pattern was observed in the results of multiple imputation analysis.

**Table 3:** Univariable regression using mean SD cut-off points for association between maternal HRQoL and development of VLBW children at 2.5 years old

Variable	Analysis Dataset No	Group	No. (%)	Physical QOL Coefficient (95% CI)	Mental QOL Coefficient (95% CI)
Communication	356	Above 1 SD below the mean [Reference]	197 (55.3%)	-	-
		Between 1–2 SD below the mean	73 (20.5%)	-1.316 (-3.346—0.714)	-0.422 (-2.096—1.251)
		Below 2 SD below the mean	86 (24.2%)	-0.825 (-2.740—1.090)	-0.994 (-2.573—0.584)
Gross motor	356	Above 1 SD below the mean [Reference]	184 (51.7%)	-	-
		Between 1–2 SD below the mean	66 (18.5%)	-1.090 (-3.218—1.038)	0.396 (-1.331—2.122)
		Below 2 SD below the mean	106 (29.8%)	-0.556 (-2.365—1.252)	-2.410 (-3.877—-0.943)
Fine motor	353	Above 1 SD below the mean [Reference]	201 (56.9%)	-	-
		Between 1–2 SD below the mean	65 (18.4%)	-0.506 (-2.629—1.617)	-0.125 (-1.860—1.610)
		Below 2 SD below the mean	87 (24.6%)	-0.543 (-2.452—1.367)	-2.145 (-3.705—-0.585)
Problem- solving	356	Above 1 SD below the mean [Reference]	205 (57.6%)	-	-
		Between 1–2 SD below the mean	53 (14.9%)	-1.086 (-3.373—1.200)	-1.482 (-3.348—0.384)
		Below 2 SD below the mean	98 (27.5%)	-0.411 (-2.233—1.412)	-1.929 (-3.416—-0.442)
Personal- social	357	Above 1 SD below the mean [Reference]	174 (48.7%)	-	-
		Between 1–2 SD below the mean	102 (28.6%)	0.349 (-1.502—2.199)	-0.783 (-2.298—0.732)
		Below 2 SD below the mean	81 (22.7%)	-0.133 (-2.129—1.863)	-1.828 (-3.462—-0.194)
Overall	353	No domain below 2 SD below the mean [Reference]	178 (50.4%)	-	-
		Any domain below 2 SD below the mean	175 (49.6%)	-0.417 (-1.999—1.165)	-1.175 (-2.476—0.125)
Overall (2 domains)	350	One or no domain below 2 SD below the mean [Reference]	244 (69.7%)	-	-
		At least 2 domains below 2 SD below the mean	106 (30.3%)	-0.326 (-2.059—1.407)	-2.260 (-3.670—-0.849)

**Table 4:** Multivariable regression using percentile cut-off points for association between maternal HRQoL and development of VLBW children at 2.5 years old

Variable	Analysis Dataset No	Group	No. (%)	Physical QOL Coefficient (95% CI)	Mental QOL Coefficient (95% CI)
Communication	262	Above 16.0 percentile [Reference]	158 (60.3%)	-	-
		Between 2.5–16.0 percentile	77 (29.4%)	-0.257 (-2.236—1.723)	0.106 (-1.360—1.572)
		Below 2.5 percentile	27 (10.3%)	-0.186 (-3.189—2.817)	-0.335 (-2.559—1.888)
Gross motor	262	Above 16.0 percentile [Reference]	140 (53.4%)	-	-
		Between 2.5–16.0 percentile	83 (31.7%)	-0.951 (-2.876—0.974)	-0.161 (-1.598—1.277)
		Below 2.5 percentile	39 (14.9%)	2.047 (-0.576—4.669)	-1.513 (-3.471—0.445)
Fine motor	259	Above 16.0 percentile [Reference]	150 (57.9%)	-	-
		Between 2.5–16.0 percentile	89 (34.4%)	0.765 (-1.162—2.693)	0.291 (-1.129—1.711)
		Below 2.5 percentile	20 (7.7%)	0.315 (-3.262—3.891)	-2.118 (-4.752—0.517)
Problem- solving	263	Above 16.0 percentile [Reference]	159 (60.5%)	-	-
		Between 2.5–16.0 percentile	67 (25.5%)	0.370 (-1.694—2.433)	-0.463 (-1.990—1.064)
		Below 2.5 percentile	37 (14.1%)	0.754 (-1.903—3.412)	-1.078 (-3.044—0.888)
Personal- social	263	Above 16.0 percentile [Reference]	167 (63.5%)	-	-
		Between 2.5–16.0 percentile	62 (23.6%)	1.542 (-0.566—3.651)	-0.517 (-2.085—1.051)
		Below 2.5 percentile	34 (12.9%)	0.930 (-1.741—3.601)	-0.077 (-2.064—1.909)
Overall	258	No domain below 2.5 percentile [Reference]	191 (74.0%)	-	-
		Any domain below 2.5 percentile	67 (26.0%)	0.759 (-1.296—2.814)	-0.345 (-1.872—1.182)
Overall (2 domains)	257	One or no domain below 2.5 percentile [Reference]	221 (86.0%)	-	-
		At least 2 domains below 2.5 percentile	36 (14.0%)	0.546 (-2.066—3.158)	-0.959 (-2.894—0.975)

**Table 5:** Multivariable regression using mean SD cut-off points for association between maternal HRQoL and development of VLBW children at 2.5 years old

Variable	Analysis Dataset No	Group	No. (%)	Physical QOL Coefficient (95% CI)	Mental QOL Coefficient (95% CI)
Communication	356	Above 1 SD below the mean [Reference]	197 (55.3%)	-	-
		Between 1–2 SD below the mean	73 (20.5%)	-1.316 (-3.346—0.714)	-0.422 (-2.096—1.251)
		Below 2 SD below the mean	86 (24.2%)	-0.825 (-2.740—1.090)	-0.994 (-2.573—0.584)
Gross motor	356	Above 1 SD below the mean [Reference]	184 (51.7%)	-	-
		Between 1–2 SD below the mean	66 (18.5%)	-1.090 (-3.218—1.038)	0.396 (-1.331—2.122)
		Below 2 SD below the mean	106 (29.8%)	-0.556 (-2.365—1.252)	-2.410 (-3.877—-0.943)
Fine motor	353	Above 1 SD below the mean [Reference]	201 (56.9%)	-	-
		Between 1–2 SD below the mean	65 (18.4%)	-0.506 (-2.629—1.617)	-0.125 (-1.860—1.610)
		Below 2 SD below the mean	87 (24.6%)	-0.543 (-2.452—1.367)	-2.145 (-3.705—-0.585)
Problem- solving	356	Above 1 SD below the mean [Reference]	205 (57.6%)	-	-
		Between 1–2 SD below the mean	53 (14.9%)	-1.086 (-3.373—1.200)	-1.482 (-3.348—0.384)
		Below 2 SD below the mean	98 (27.5%)	-0.411 (-2.233—1.412)	-1.929 (-3.416—-0.442)
Personal- social	357	Above 1 SD below the mean [Reference]	174 (48.7%)	-	-
		Between 1–2 SD below the mean	102 (28.6%)	0.349 (-1.502—2.199)	-0.783 (-2.298—0.732)
		Below 2 SD below the mean	81 (22.7%)	-0.133 (-2.129—1.863)	-1.828 (-3.462—-0.194)
Overall	353	No domain below 2 SD below the mean [Reference]	178 (50.4%)	-	-
		Any domain below 2 SD below the mean	175 (49.6%)	-0.417 (-1.999—1.165)	-1.175 (-2.476—0.125)
Overall (2 domains)	350	One or no domain below 2 SD below the mean [Reference]	244 (69.7%)	-	-
		At least 2 domains below 2 SD below the mean	106 (30.3%)	-0.326 (-2.059—1.407)	-2.260 (-3.670—-0.849)

### Association between child development and maternal HRQoL by the presence or absence of subjective social connection and cooperation of partner

**Fig. 2:**
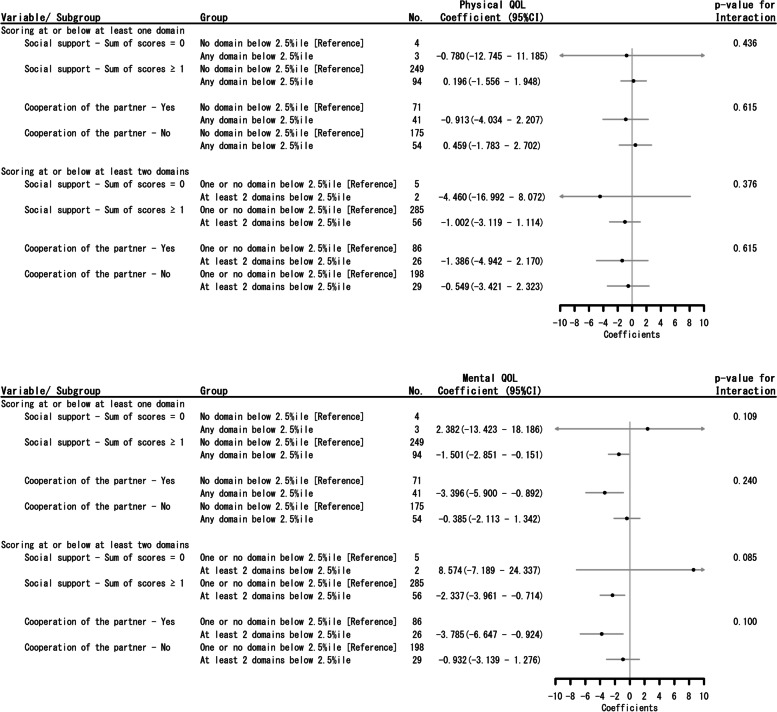
**1** Univariate regression of the association between social connection / cooperation of the partner and physical HRQoL by probability of scoring “Needs assessment” on J-ASQ-3 (The arrows of confidence intervals indicate the wide range of upper or lower limit.). 2: Univariate regression of the association between social connection / cooperation of the partner and mental HRQoL by probability of scoring “Needs assessment” on J-ASQ-3 (The arrows of confidence intervals indicate the wide range of upper or lower limit.)

The results of univariable regression analysis show that there was no association between maternal physical HRQoL and the status of child development by the presence or absence of subjective social connection and cooperation of partner (Fig. 2-1). Amongst women who indicated having some social support, having a child with a SDD in one domain or in two or more domains was negatively associated with mental HRQoL compared with women whose child was less developmental delay (regression coefficient –1.501 [95%CI: –2.851 to –0.151] and regression coefficient –2.337 [95%CI: –3.961 to –0.714]). In addition, having a child with a SDD in one domain or in two or more domains was negatively associated with mental HRQoL among women who reported having partner’s cooperation to child-rearing, compared with women whose child was less developmental delay (regression coefficient –3.396 [95%CI: –5.900 to –0.892] and regression coefficient –3.785 [95%CI: –6.647 to –0.924]). (Fig. 2-2). There were no statistically significant subgroup interactions in both physical and mental HRQoL.

## Discussion

The present study described the prevalence of SDDs in Japanese VLBW infants. The developmental delay in at least two domains below 2.5 percentile was 16.2%, which is almost the same as the prevalence of developmental delay at 3 years of age (16.3%) reported in the largest Japanese registry of VLBW infants [2]. Although the ASQ-3 was originally developed to monitor premature infants discharged from NICUs [6] and is one of the most frequently used screening measures that has a body of evidence [29], there are few reports on VLBW children evaluated with the ASQ-3 [30, 31]. To our knowledge, no previous study was available to compare the prevalence rates of SDDs at 2.5 years with VLBW using cut-off of ASQ-3 scores below 2.5 percentile. Schonhaut et al. [32] reported that 24% of preterm born children at 2.5 years in Chile had delay in any domain of the ASQ-3 screened below 2 SD below the mean score, which was much lower than our result of SDDs (49.6%) below 2 SD below the mean score. The distribution of ASQ-3 scores may differ depending on country and language as pointed out by cross-cultural examinations [33, 34]. Furthermore, Valla et al. [35] claimed that the previous studies have shown substantial variations in the prevalence of developmental delay because of methodological issues, including differences in case definition and criteria, type of measures used, and age range. In their study, the prevalence of SDDs was estimated at 4–12 months in the general population using the US cut-off (below 2 SD below the mean) and the Norwegian cut-off (below 2 percentile) of the ASQ-3. The prevalence of SDDs scoring at or below the cut-off of 2 SD below the mean in at least one developmental area was higher than the cut-off of below 2 percentiles in all age groups. This corresponds to our findings and indicates that it is important to avoid thetic interpretation on the prevalence of SDDs in a non-clinical setting.

The lower mental HRQoL was significantly associated with SDDs in any domain and at least two domains, whereas no association was found with physical HRQoL. It is understandable that anxiety and distress associated with SDDs have greater impact on mental wellbeing rather than the physical burden of mothers. Although the multivariate regression analysis in our study showed no statistically significant associations, the worse the degree of child development the lower the maternal HRQoL coefficients. This also supports the concept that maternal HRQoL may be influenced by the degree of child development. Previous studies examined the maternal HRQoL of preterm or VLBW infants in comparison with term infants and those results were inconsistent [11, 13, 14]. Our study extended those findings by comparing the degree of development in VLBW infants and explored the possibilities that maternal HRQoL is related not only to maturity and birth weight, but also to the degree of child development. The present study focused on maternal HRQoL, which might respond differently from their partner’s or both parent’s HRQoL given the strong influence of gender norms such as roles required in child-rearing.

The high percentage of mothers who always received their partner’s cooperation and the very few mothers who responded no social connection were favorable results. As participants in the JECS who have health issues or were born immature tend to drop out from the survey, it is conceivable that the continuing participants are highly interested in environmental health issues and parenting. This may reflect the high percentages of having social connection and partner’s cooperation in our population. Interestingly, mothers who responded having social connection and partner’s cooperation and had children with SDDs in any domain and at least two domains had significantly lower mental HRQoL than mothers of children without SDD. This is inconsistent with our hypothesis that the lack of social connection and the lack of support from partners lowers the maternal HRQoL, regardless of the degree of child development. The reason for this finding is uncertain; however, a study with a large sample size with more detailed information regarding social connection and partnerships could elucidate this contradiction.

According to the survey on the municipal maternal and child health activities conducted by the Japanese Ministry of Health, Labour and Welfare in 2013, 83.1% of municipalities (54 out of 65) used the Health Guidance Manual for Low Birth Weight Infants to provide basic knowledge for guidance and to support healthy child-rearing by reducing anxiety when implementing the medical supporting services and home visit program for the premature babies and their caregivers [36]. The survey reported that a high percentage of guidance and support was provided for the child’s development and illness, follow-up outpatient visits, information on medical care and rehabilitation as well as for the mother’s child-rearing anxiety and mental health. Early intervention and support seem to be well provided in the current schemes. Our findings suggest that it is important to provide continued support and regular assessment to detect developmental delay for mothers of VLBW children.

This may be the first study to investigate the association between maternal HRQoL and development of VLBW children using the J-ASQ-3 in a large epidemiological cohort from the Japanese general population. One of the strengths of this study is that the number of study subjects is relatively large compared with previous research for this high-risk pediatric population. On the other hand, the present study has several limitations. First, we were not able to include the days of NICU hospitalization as a co-variate due to the high percentage of missing data. The longer stay in the NICU, the more likely is the severe health condition of the child. The length of NICU hospitalization may impact on physical and mental well-being of mothers after discharge [12, 37] and in early childhood [13]. Secondly, mothers who had difficulty responding to the self-administered survey (e.g., due to developmental disabilities) were excluded in this study. Maternal developmental disorders, for example, can affect the degree of child development and maternal HRQoL. Thirdly, although the current study is part of the cohort study collecting data from the early pregnancy for every six months, the time point of assessing both maternal HRQoL and child development was only at 2.5 years of age in the currently available data. If we collected both variables at several time points, we could have examined a trajectory of child development and maternal HRQoL. Finally, screening child development using parent-completed questionnaires such as ASQ-3 may be affected by parent’s values or wishes because there is no standard manual to refer when answering the questions. Heiser et al. [38] reported that parents of VLBW infants tend to underestimate their infant’s development and described the tendency of VLBW parent’s anxiety that may cause over-rating of minor differences. This may also apply to this study population.

## Conclusions

The present study examined the associations between the maternal HRQoL and child development of VLBW children at 2.5 years of age. We found that the lower mental HRQoL was independently associated with the SDDs evaluated by the J-ASQ-3, whereas there was no association after adjusting for co-variates. Further research is warranted to elucidate the impact of social connection and partner’s cooperation on maternal HRQoL and child development. This study urges that particular attention should be paid to mothers of VLBW children with SDDs and also to provide early intervention and continued support.

## Supplementary Information

**Additional file 1: Supplementary Table 1. **Summary of Categorical J-ASQ - Percentile Method (*N* = 357)

**Additional file2: Supplementary Table 2. **Summary of Categorical J-ASQ - Gaussian Method (*N* = 357)

## Data Availability

The datasets generated and/or analysed during the current study are not publicly available due to ethical restrictions and legal framework of Japan. It is prohibited by the Act on the Protection of Personal Information (Act No. 57 of 30 May 2003, amendment on 9 September 2015) to publicly deposit the data containing personal information. Ethical Guidelines for Medical and Health Research Involving Human Subjects enforced by the Japan Ministry of Education, Culture, Sports, Science and Technology and the Ministry of Health, Labour and Welfare also restricts the open sharing of the epidemiologic data. All inquiries about access to data should be sent to: jecs-en@nies.go.jp. The person to be contacted for study data sent to this e-mail address is Dr Shoji F. Nakayama, the Deputy General Manager of the Japan Environment and Children’s Study (JECS) Programme Office, National Institute for Environmental Studies.

## Abbreviations

ASQ-3: Ages and Stages Questionnaire, 3^rd^ edition
GA: Gestational age
HRQoL: Health-related quality of life
NICU: Neonatal intensive care unit
VLBW: Very low birth weight
