# Supplementary material for: Health-related quality of life of mothers and developmental characteristics of very low birth weight children at 2.5 years of age: results from the Japan Environment and Children’s Study (JECS)
Source: Health Qual Life Outcomes. 2023 Jul 10;21:68. doi: 10.1186/s12955-023-02156-4 (PMC10331955; doi:10.1186/s12955-023-02156-4)
Supplement: Supplementary file 1 — Additional file 1: Supplementary Table 1. Summary of Categorical J-ASQ - Percentile Method (N = 357) [file 12955_2023_2156_MOESM1_ESM.doc]

Supplementary Table 1

Summary of Categorical J-ASQ - Percentile Method (N = 357)


Communication (Cut off: 15, 45)

C2hy_ASQ_aComu_cate	Frequency	Proportion	
1: normal (≥ 45)	197	55.2%	
2: monitoring (≥ 15, < 45)	117	32.8%	
3: need assessment (< 15)	42	11.8%	
Missing	1	0.3%	


Gross Motor (Cut off: 30, 50)

C2hy_ASQ_bGM_cate	Frequency	Proportion	
1: normal (≥ 50)	183	51.3%	
2: monitoring (≥ 30, < 50)	116	32.5%	
3: need assessment (< 30)	57	16.0%	
Missing	1	0.3%	


Fine Motor (Cut off: 15, 35)

C2hy_ASQ_cFM_cate	Frequency	Proportion	
1: normal (≥ 35)	201	56.3%	
2: monitoring (≥ 15, < 35)	117	32.8%	
3: need assessment (< 15)	35	9.8%	
Missing	4	1.1%	


Problem Solving (Cut off: 20, 40)

C2hy_ASQ_dProblem_cate	Frequency	Proportion	
1: normal (≥ 40)	205	57.4%	
2: monitoring (≥ 20, < 40)	93	26.1%	
3: need assessment (< 20)	58	16.2%	
Missing	1	0.3%	


Personal - Social (Cut off: 25, 40)

C2hy_ASQ_ePersonal_cate	Frequency	Proportion	
1: normal (≥ 40)	216	60.5%	
2: monitoring (≥ 25, < 40)	89	24.9%	
3: need assessment (< 25)	52	14.6%	
