# Supplementary material for: Health-related quality of life of mothers and developmental characteristics of very low birth weight children at 2.5 years of age: results from the Japan Environment and Children’s Study (JECS)
Source: Health Qual Life Outcomes. 2023 Jul 10;21:68. doi: 10.1186/s12955-023-02156-4 (PMC10331955; doi:10.1186/s12955-023-02156-4)
Supplement: Supplementary file 2 — Additional file2: Supplementary Table 2. Summary of Categorical J-ASQ - Gaussian Method (N = 357) [file 12955_2023_2156_MOESM2_ESM.doc]

Supplementary Table 2Additional file 1

Summary of Categorical J-ASQ - Gaussian Method (N = 357)


Communication (Cut off: 29.43, 41.13)

ASQ	Frequency	Proportion	
1: normal (≥ 41.13)	197	55.2%	
2: monitoring (≥ 29.43, < 41.13)	73	20.4%	
3: need assessment (< 29.43)	86	24.1%	
Missing	1	0.3%	


Gross Motor (Cut off: 37.57, 46.10)

ASQ	Frequency	Proportion	
1: normal (≥ 46.10)	184	51.5%	
2: monitoring (≥ 37.57, < 46.10)	66	18.5%	
3: need assessment (< 37.57)	106	29.7%	
Missing	1	0.3%	


Fine Motor (Cut off: 21.71, 34.38)

ASQ	Frequency	Proportion	
1: normal (≥ 34.38)	201	56.3%	
2: monitoring (≥ 21.71, < 34.38)	65	18.2%	
3: need assessment (< 21.71)	87	24.4%	
Missing	4	1.1%	


Problem Solving (Cut off: 26.54, 38.49)

ASQ	Frequency	Proportion	
1: normal (≥ 38.49)	205	57.4%	
2: monitoring (≥ 26.54, < 38.49)	53	14.8%	
3: need assessment (< 26.54)	98	27.5%	
Missing	1	0.3%	


Personal - Social (Cut off: 29.87, 40.01)

ASQ	Frequency	Proportion	
1: normal (≥ 40.01)	174	48.7%	
2: monitoring (≥ 29.87, < 40.01)	102	28.6%	
3: need assessment (< 29.87)	81	22.7%	
